# Telomere length and outcome of treatment for pulmonary tuberculosis in a gold mining community

**DOI:** 10.1038/s41598-021-83281-2

**Published:** 2021-02-17

**Authors:** Patrick D. M. C. Katoto, Tony Kayembe-Kitenge, Krystal J. Godri Pollitt, Dries S. Martens, Manosij Ghosh, Jean B. Nachega, Benoit Nemery, Tim S. Nawrot

**Affiliations:** 1 Department of Public Health and Primary Care, Centre for Environment and Health, KU Leuven, Leuven, Belgium; 2 Department of Internal Medicine, Division of Respiratory Medicine, CEGEMI and Prof. Lurhuma Biomedical Research Laboratory, Mycobacterium Unit, Catholic University of Bukavu, Bukavu, Democratic Republic of Congo; 3 Department of Medicine and Center for Infectious Diseases, Faculty of Medicine and Health Sciences, Stellenbosch University, Cape Town, South Africa; 4 Department of Public Health, Unit of Toxicology, University of Lubumbashi, Lubumbashi, Democratic Republic of Congo; 5 Department of Environmental Health Sciences, School of Public Health, Yale University, New Haven, CT USA; 6 Centre of Environmental Health, University of Hasselt, Agoralaan gebouw D, 3590 Diepenbeek, Belgium; 7 Departments of Epidemiology and International Health, Johns Hopkins Bloomberg School of Public Health, Baltimore, MD USA; 8 Departments of Epidemiology, Infectious Diseases and Microbiology, University of Pittsburgh Graduate School of Public Health, Pittsburgh, PA USA

**Keywords:** Biomarkers, Health occupations, Diseases, Infectious diseases, Tuberculosis

## Abstract

Telomere length (TL) is a marker of ageing and mitochondrial DNA (mtDNA) is an early marker of inflammation caused by oxidative stress. We determined TL and mtDNA content among active pulmonary tuberculosis (PTB) patients to assess if these cellular biomarkers differed between artisanal miners and non-miners, and to assess if they were predictive of treatment outcome. We conducted a prospective cohort study from August 2018 to May 2019 involving newly diagnosed PTB patients at three outpatient TB clinics in a rural Democratic Republic of Congo. We measured relative TL and mtDNA content in peripheral blood leukocytes (at inclusion) via qPCR and assessed their association with PTB treatment outcome. We included 129 patients (85 miners and 44 non-miners) with PTB (median age 40 years; range 5–71 years, 22% HIV-coinfected). For each increase in year and HIV-coinfection, TL shortened by − 0.85% (− 0.19 to − 0.52) (p ≤ 0.0001) and − 14% (− 28.22 to − 1.79) (p = 0.02) respectively. Independent of these covariates, patients with longer TL were more likely to have successful TB treatment [adjusted hazard ratio; 95% CI 1.27 for a doubling of leucocyte telomere length at baseline; 1.05–1.44] than patients with a shorter TL. Blood mtDNA content was not predictive for PTB outcome. For a given chronological age, PTB patients with longer telomeres at time of diagnosis were more likely to have successful PTB treatment outcome.

## Introduction

Pulmonary tuberculosis (PTB) kills almost 2 million individuals every year and is thus a leading cause of death among adults worldwide. *Mycobacterium tuberculosis* (Mtb) infects more than 10 million people each year^1–3^. Mtb potently induces cytokines and chemokines from polymorphonuclear cells and monocytes, thus resulting in intense local inflammation in the lungs^4^. Alveolar macrophages are anti-inflammatory in nature, but their function can be impaired by pollutants, including mineral dusts, thereby diminishing the body’s ability to clear infections^5–8^. This is probably why mineworkers are more susceptible to develop PTB.

Telomere length (TL) reflects the history of oxidative stress and chronic inflammation, and is a marker for age-related disease susceptibility^9–12^. In normal physiology, mitochondria are important in the cell as they generate most of the adenosine triphosphate (ATP) through the oxidative phosphorylation mechanism (OXPHOS), which is a critical energy supply for cellular processes and partially encoded with mitochondrial DNA (mtDNA). The OXPHOS mechanism uses dietary intake to produce ATP, but it also produces ROS which can destroy mitochondrial DNA, impairs respiratory chain function and cause nuclear DNA damage^12–14^. Further, Mitochondrial DNA (mtDNA) damage can result in genomic instability, cellular senescence and altered intercellular communication. Hallmarks of aging genomic instability and deregulated nutrient sensing can contribute to reduced mitochondrial dynamics, while reduced mitochondrial dynamics can cause cellular senescence^15^. Decreased mitochondrial function, with impaired ATP generation, is considered, together with TL, as belonging to the core axis of ageing, which might be influenced by environmental conditions^16^. In view of their sensitivity to oxidative DNA alteration and inflammation, TL and mitochondrial DNA content (mtDNA) can be viewed as cellular sensors of stress following exposure to environmental pollutants, and biomarkers of vulnerability to chronic infectious diseases. These biological markers of ageing, therefore, may predict disease outcome^17–22^.

We aimed to determine TL and mtDNA content in persons with documented active PTB in a gold-mining area of the eastern Democratic Republic of Congo (DRC) to assess if these cellular biomarkers differed between artisanal miners and non-miners, and to assess if they were predictive of treatment outcome.

## Results

### Characteristics of study participants

**Figure 1:**
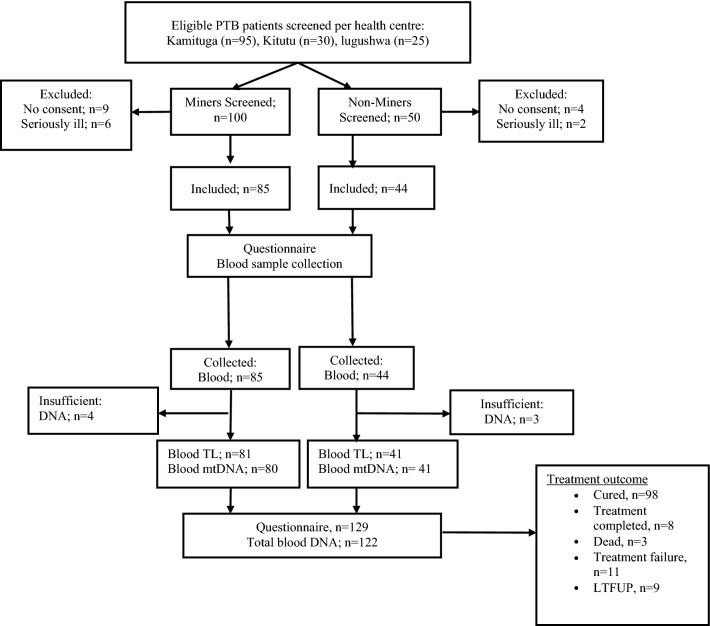
Study flow chart. *PTB* pulmonary tuberculosis, *TL* telomere length, *mtDNA* mitochondrial DNA.

Figure 1 displays the study flow chart. Tables 1 and 2 show baseline characteristics of the 129 PTB patients enrolled in the study by mining related activities and by treatment outcomes, respectively. Their age ranged from 5 to 71 years with an overall median age of 40 years (IQR 31–49 years). This includes eight children below 14 years belonged to the same household as an adult study participant. BMI was very low (median 16) reflecting active PTB disease and undernutrition. Of the 129 included participants, 28 (22%) were HIV-infected, 19 (15%) were re-treated TB cases and eight patients (6%) were RR-TB. Forty-four participants (52% females) had never worked in mining and were labelled “non-miners”; 85 participants (44% females) had a history of work as miners or close involvement in mining-related activities (including three children of adult participants) and they were all considered to be “miners”. Miners and non-miners had quasi-similar socio-demographic and clinical characteristics.

**Table 1:** Baseline characteristics of 129 PTB patients working or living around artisanal mining of gold in Eastern DR Congo.

Variables	Total	Non-miners	Miners^a^	p value
	(n = 129)	(n = 44)	(n = 85)	
**Age (years)**				
Median (IQR)	40 (31–49)	40 (26–49.5)	40 (33–48)	0.59
**Sex**				
Female	60 (47%)	23 (52%)	37 (44%)	0.35
Male	69 (53%)	21 (48%)	48 (56%)	
**BMI (kg/m**^**2**^**)**				
Median (IQR)	16 (15–18)	16 (15–17)	16 (15–18)	0.73
**Work in mining (years)**				
Median (IQR)	–	–	10 (5–15)	–
**Tobacco smoking**				
No	116 (91%)	41 (93%)	75 (90%)	0.74
Yes	11 (9%)	3 (7%)	8 (10%)	
**Xpert MTB/RIF (+)**				
No	121 (94%)	41 (93%)	80 (94%)	0.83
Yes	8 (6%)	3 (7%)	5 (6) %	
**New case**				
No	19 (15%)	3 (7%)	16 (19%)	0.06
Yes	109 (85%)	41 (93%)	68 (81%)	
**HIV-infection**				
No	101 (78%)	36 (82%)	65 (77%)	0.48
Yes	28 (22%)	8 (18%)	20 (24%)	
**Telomere length**^**b**^				
Median (IQR)	0.99 (0.88–1.14) (n = 122)	1.05 (0.89–1.26) (n = 41)	0.97 (0.84–1.14) (n = 81)	0.22
**mtDNA**^**c**^				
Median (IQR)	1.04 (0.82–1.21) (n = 121)	1.05 (0.81–1.21) (n = 41)	0.99 (0.84–1.15) (n = 80)	0.52

Data are shown as n (%) otherwise as median and interquartile range (IQR).

*BMI* body mass index, *Xpert MTB/RIF* automated diagnostic test for rapid identification of *Mycobacterium tuberculosis* DNA and resistance to rifampicin (RIF).

^a^Miners are defined as participants reporting current or recent work in mining or ancillary activities.

^b^Leukocyte TL is expressed in relative units as the ratio of telomere copy number was proportional to single-copy gene number (T/S) relative to the average telomere length and mtDNA content respectively. T/S ratio of the entire sample set (n = 122).

^c^Leukocyte mtDNA is expressed in relative units as the ratio of ND1 copy number to single-copy gene number relative to the average ratio of the entire sample set (n = 121).

p-values refer to difference between non-miners and miners (without adjustments).

**Table 2:** Characteristics at inclusion of 129 PTB patients according to treatment outcome.

Variables	Declared cured		p-values
	Yes (n = 98)	No (n = 31)	
**Age (years)**			
Median (IQR)	40 (31–48)	45 (34–50)	0.22
**Sex**			
Female	50 (51%)	10 (32%)	0.07
Male	48 (49%)	21 (68%)	
**BMI (kg/m**^**2**^**)**			
Median (IQR)	16 (15–18)	17 (15–17)	0.37
**Work in mining**			
No	33 (34%)	11 (36%)	0.85
Yes	65 (66%)	20 (64%)	
**Work in mining (years)**			
Median (IQR)	10 (5–15)	12 (9–15)	0.26
**Tobacco smoking**			
No	88 (8%)	28 (90%)	0.81
Yes	8 (8%)	3 (10%)	
**Xpert MTB/RIF (+)**			
No	92 (94%)	29 (94%)	0.94
Yes	6 (6%)	2 (6%)	
**New case**			
No	14 (14%)	5 (17%)	0.74
Yes	84 (86%)	25 (83%)	
**HIV-infection**			
No	82 (84%)	19 (61%)	0.008
Yes	16 (16%)	12 (39%)	
**Telomere length**^**a**^			
Median (IQR)	1.04 (0.90–1.19) (n = 92)	0.92 (0.77–1.04) (n = 30)	0.02
**mtDNA**^**b**^			
Median (IQR)	1.08 (1.02–1.21) (n = 92)	0.99 (0.81–1.20) (n = 29)	0.18

Data are shown as n (%) otherwise as median and interquartile range (IQR).

*BMI* body mass index, *Xpert MTB/RIF* automated diagnostic test for rapid identification of *Mycobacterium tuberculosis* DNA and resistance to rifampicin (RIF).

^a^Leukocyte TL and mtDNA is expressed in relative units as the ratio of telomere copy number was proportional to single-copy gene number (T/S) relative to the average telomere length and mtDNA content respectively. T/S ratio of the entire sample set (n = 122).

^b^Leukocyte mtDNA is expressed in relative units as the ratio of ND1 copy number to single-copy gene number relative to the average ratio of the entire sample set (n = 121).

p-values refer to difference between declared cured vs not declared cured (without adjustments).

For outcomes assessment, we verified the clinical registries after completion of TB treatment, i.e. between 6 and 9 months after inclusion in the cohort. About 80% of patients were declared cured, with no differences between miners and non-miners (Table 1). Of the 31 patients (20 miners) who were not declared cured, three had died (two miners), 11 were treatment failures (seven miners), nine were lost to follow-up or transferred-out (eight miners) and eight were treatment completers (three miners). Although the proportion of females tended to be higher among patients declared cured (p < 0.07), demographic, clinical or occupational characteristics did not differ significantly between patients with successful treatment and those without successful treatment, except for HIV-infection, which was more prevalent (p = 0.008) among patients without successful treatment (39%) than among those declared cured (16%).

### Markers of biological ageing

**Figure 2:**
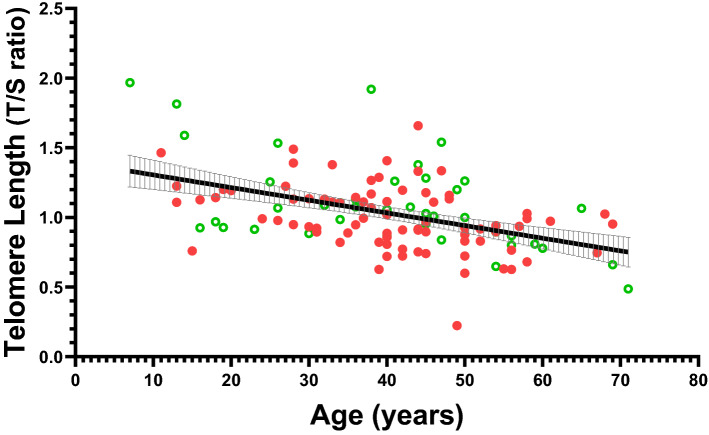
Relation between leukocyte TL and age among 122 participants with pulmonary tuberculosis, of whom 81 were miners (full symbols) and 41 were non-miners (open symbols). The crude Pearson’s regression line is shown (with 95% confidence interval) and has a slope of − 0.009 (95% CI − 0.012 to − 0.006); R^2^ = 0.21. Leukocyte TL is expressed in relative units as the ratio of telomere copy number to single-copy gene number (T/S) relative to the average T/S ratio of the entire sample set (n = 122).

Figure 2 shows that, consistent with theory, TL was shorter with increasing age in both miners and non-miners. No such difference was observed for mtDNA. Furthermore, Table 3 shows that TL was independently associated with age [Percent difference (95% CI)] − 0.85% per year increase in age (− 0.19 to − 0.52%) (p ≤ 0.0001) as well as independently associated with HIV-coinfection [Percent difference (95% CI)] − 14% (− 28.22 to − 1.79%) (p = 0.02). Further, miners tended to have a shorter TL (p = 0.05) (Table 3 and Supplentary Table 1). Blood mtDNA content was only significantly associated with BMI (Table 3).

**Table 3:** Multivariable linear regression analysis of predictors for biomarkers of ageing among patients with pulmonary tuberculosis.

Variables	Leukocyte telomere length		Mitochondrial DNA content	
	Percent difference (95% CI)	p-value	Percent difference (95% CI)	p-value
Age (+ 1 year)	− 0.85 (− 1.19 to − 0.52)	< 0.0001	− 0.21 (− 0.55 to 0.14)	0.24
**Sex**				
Female	Ref			
Male	6.07 (− 3.2 to 14.51)	0.19	1.02 (− 8.31 to 11.32)	0.83
BMI (+ 1 kg/m^2^)	1.25 (− 1.22 to 3.78)	0.32	2.57 (0.05–5.24)	0.05
**Mining-related activities**				
No	Ref			
Yes	− 9.99 (− 21.15 to 0.14)	0.05	3.85 (− 5.96 to 14.68)	0.45
**Tobacco smoking**				
No	Ref			
Yes	− 11.87 (− 31.64 to 4.94)	0.18	− 14.67(− 27.72 to 0.76)	0.06
**Tuberculosis category**				
New case	Ref			
Retreatment	− 7.11 (− 22.06 to 6.01)	0.30	1.38 (− 11.35 to 15.95)	0.84
**HIV (+)**				
No	Ref			
Yes	− 14.24 (− 28.22 to − 1.79)	0.02	− 4.72 (− 15.5 to 7.43)	0.43

All models are adjusted for sociodemographic (age, gender, etc.) and clinical variables (BMI, HIV status, smoking history, mining related activity status).

*BMI* body mass index, *Ref.* reference.

### Biological ageing and successful tuberculosis treatment

TL measured at inclusion, was significantly longer (p < 0.02, Mann–Whitney test) in the 92 participants who, six to nine months later, were declared cured (TL 1.04 [0.90–1.19]) than in the 30 participants who were not declared cured (TL 0.92 [0.77–1.04]) (Table 2). This was confirmed in multiple Cox regression analysis (Table 4) which indicated that, independently of sociodemographic such as age and gender and clinical variables included in the model, participants with longer TL at inclusion were more likely to be declared cured at follow-up 6 to 9 months later.

**Table 4:** Multivariable cox regression analysis of predictors associated with pulmonary tuberculosis treatment success among artisanal miners and relatives.

Treatment success						
Variables	Model 1		Model 2			
	All participants (n = 122)		Non-miners (n = 41)		Miners (n = 81)	
	aHR (95% CI)	p-value	aHR (95% CI)	p-value	aHR (95% CI)	p-value
**Mining-related activities**						
No	Ref					
Yes	1.17 (0.84–1.63)	0.35	–	–	–	–
Doubling of TL	1.27 (1.05–1.44)	0.019	1.32 (0.86–1.59)	0.14	1.36 (1.14–1.52)	0.003
Doubling of mtDNA content	1.11 (0.65–1.88)	0.65	1.17 (0.64–2.18)	0.61	1.24 (0.48–3.21)	0.65

Hazard ratios and p-values were computed by Cox regression and expressed for a doubling in TL and mtDNA content at baseline. Model 1 includes all participants. Model 2 stratifies participants by mining related-activities. All models are adjusted for age, sex, tobacco smoking, TB category, years working in artisanal mining (when applicable), HIV-infection status and body mass index. Model 1 also adjusted for Xpert MTB/RIF result.

*TL* leukocyte telomere length, *mtDNA* mitochondrial DNA, *aHR* adjusted hazards ratio, *Ref.* reference.

**Figure 3:**
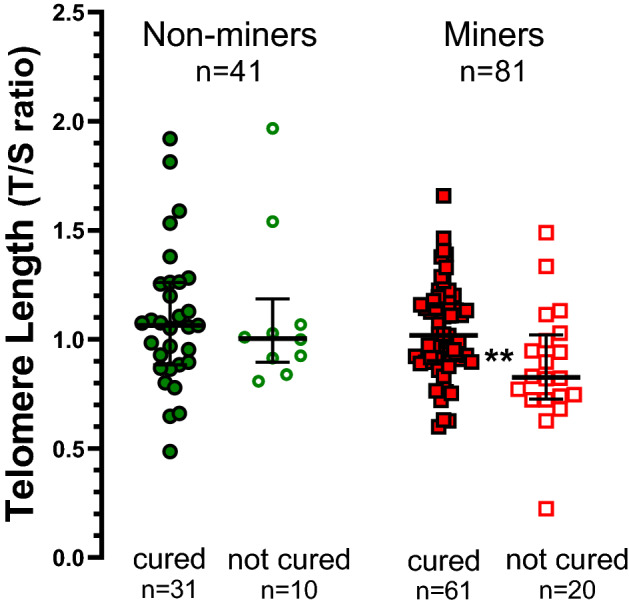
Leukocyte telomere length measured at inclusion among 122 participants with pulmonary tuberculosis, of whom 41 were non-miners (left) and 81 were miners (right); six to nine months later, the patients were evaluated and divided into “cured” and “not crude”. Data are individual values and medians with 25th and 75th percentiles. Among miners, TL length was significantly shorter (**p < 0.01 by Mann Whitney test) among patients not declared cured than among cured patients.

The treatment success associated with a doubling of telomere length at baseline was 1.27 (95% CI 1.05–1.44]. However, as shown in Fig. 3 and Table 4, after stratification according to mining status, this effect appeared to be present only among the larger group of TB patients with a history of mining (aHR 1.36; 95% CI 1.14–1.52). mtDNA content was not associated with successful TB treatment, neither overall, nor after stratification by mining status (Table 4).

## Discussion

In this artisanal and small-scale gold mining community, we investigated if artisanal miners were more likely to have worse clinical presentations and/or poorer outcomes of PTB than non-miners from the same area. This proved not to be the case: patients with a history of working in gold mining did not differ from non-miners and they were not significantly more likely to fail treatment. However, in our population of patients with PTB in general, we did find that short leukocyte TL, a biomarker of ageing, measured during the active phase of treatment shortly after diagnosis of TB, was associated with TB treatment failure, as assessed after TB treatment completion. This association was independent of age and HIV status (both of which were associated with a shorter TL), smoking and relevant clinical variables, including MDR-TB (present in 6% of the studied population). No such association was observed for mtDNA content.

We investigated, for the first time, the association between TL or mtDNA content and TB treatment outcome. This study does not only generate data from this remote rural African region but also increases our knowledge of the relationship between ageing biomarkers and the deadliest infectious disease worldwide. In agreement with studies in other populations^19,22–25^, we found that TL shortens over the life course. The prevalence of smoking was low (9%) and, also consistent with the literature^17,24,26^, TL tended to be shorter among smokers. mtDNA was not associated with any variable except with BMI, which was very low (median of 16 kg/m^2^) in our population of TB patients, but the significantly positive association between BMI and mtDNA suggests that mitochondrial injury (low mtDNA) reflects more severe disease and/or undernutrition. While telomere shortening has been associated with increased risk for chronic disease, there has been less research on the possible relation between TL and prognosis of acute diseases or response to treatment. In line with previous studies^24,25,27,28^ we found shorter leukocyte TL in HIV-positive participants, thus supporting an acceleration of cell senescence during HIV infection. In a previous study in HIV patients, PTB was not independently associated with TL^24^ but that study included patients hospitalized for TB suspicion and it only evaluated mortality after two months. In the same cohort^29^, the known genetic sum score of TL appeared to be associated with TL in the TB-negative group, but not in the TB-positive group. In a case–control study in Thailand, TB patients had lower TL compared with controls, but in TB patients longer telomere length was associated with anti-tuberculosis drug-induced liver injury^30^.

The estimate between telomere length and treatment success in non-miners (odds ratio 1.32) and miners (odds ratio 1.36) did not differ much (Table 4). The wider confidence interval of the aforementioned association in the group of non-miners can be explained by the lower power as this group included only 41 persons. Our association between TL and treatment success can be explained via two pathways. First, persons with longer telomeres might have better immune competency^31^ which might explain the successful treatment of PTB in persons with longer telomere length. Second, alternatively we can speculate that short TL is reflective of the intensity or duration of past inflammation and tissue damage caused by TB. The acute-phase response in active PTB is due to inflammation, infection or tissue injury and is characterized by cytokine-induced release into the circulation of proteins predominantly synthesized in the liver. We know that TL captures historical inflammatory conditions and it has been shown that CRP concentrations may correlate with therapeutic response^9,12,18,19^. In other words, our PTB patients with shorter TL at baseline might reflect a longer history of TB and/or more severe PTB. Although we had newly diagnosed cases, we cannot exclude that those with shorter TL had been ill for a longer time.

Among limitations to our study is the convenience sampling technique used to obtain the study population, which may have entailed a recruitment bias, but we had no other option in this remote setting. Of note, although described as “post-conflict,” the security situation in eastern DRC is still precarious because of the continued presence of active militias, especially around mining areas. Nevertheless, despite challenging circumstances, we have been able to obtain reliable clinical information on TB (and HIV) from existing hospital registries (both laboratory and treatment outcome) and double check them via the district registry of TB. Another limitation is our limited sample size, so we cannot exclude the possibility of a type 2 error that, together with unmeasured confounding factors, could account for the negative finding of our primary objective.

In conclusion, our findings show that independently of chronological age, TL in TB patients was associated with (and, hence, possibly determined) the success of pharmacological interventions. Whether this is because TL reflects disease severity or because blood TL in TB patients is a marker of immune senescence (or both) will require further clinical studies.

## Methods

### Study design, settings and participants

In three one-day campaigns (from 1st to 3rd of August 2018), we established a cohort of 129 patients with recently confirmed PTB (Fig. 1), who were all included during the first two weeks of their two-month intensive treatment phase under the National TB Program (NTP). Due to insecurity in the mining region, it was not possible to recruit TB patients from villages or mining sites by systematic sampling, and we resorted to convenience sampling from three health facilities (Kamituga, Kitutu, and lugushwa) situated in a remote rural area with (underground) artisanal and small-scale mining of gold in the South Kivu province, eastern DRC. Participants were patients (adults and their children) attending scheduled clinic visits for Directly Observed Treatment (DOT) and patients invited by mobile phone to join the study. Clinical data, occupational history and blood (and urine) were obtained at recruitment. Between six and nine months after recruitment, i.e. after completion of the treatment, we assessed the outcome based on the clinical records using existing hospital registries (both laboratory and treatment outcome) and double check them via the district registry of TB.

### Pulmonary TB: case definition, treatment regimen and clinical outcome definition

Existing medical records (laboratory and treatment registries) were used, at both inclusion and follow-up, to ascertain TB-specific data (biologic characteristics, HIV-coinfection, TB treatment regimen, and clinical outcome). Patients were diagnosed and managed according to the Congolese NTP and described previously^32,33^. In brief, the diagnosis of PTB was based on a compatible clinical history with confirmation by *Ziehl–Neelsen* sputum smear microscopy for acid-fast bacilli (AFB) and Xpert performed on different sputum samples. Chest radiography was not available in the area. The standard 6-month first-line drugs (FLDs) regimen, i.e., 2 months of rifampicin (*R*), isoniazid (*H*), pyrazinamide (*Z*), and ethambutol (*E*) followed by 4 months of *R* and *H* (*2RHZE/4RH*) were used to treat new cases, rifampicin-susceptible patients-TB (RS-TB). An 8-month-based regimen (*2SRHZE/1RHZE/5RHE*) including streptomycin (*S*) for the first 2 months was used to manage patients retreated after failure, relapsed, or lost to follow-up (LTFU). Patients with rifampicin-resistant-TB (RR-TB) received the standard 9-month shorter regimen with moxifloxacin (*Mfx*), clofazimine (*Cfz*), *E,* and *Z* throughout, supplemented by kanamycin (*Km*), prothionamide (*Pto*), and high-dose isoniazid (*H*_*h*_) during a 4-month intensive phase (*4 Km–Mfx–Pto–Cfz–Z–H*_*h*_*–E/5 Mfx–Cfz–Z–E*) Patients were managed on an outpatient basis, and DOT was supervised by a nurse at the nearest TB diagnostic and treatment centres, either daily during the intensive phase or weekly during the continuation phase, with trained community health workers or an assigned family member observing TB treatment at home on the remaining days of the week.

### Clinical outcome definition

Outcomes were defined according to the WHO definitions^34^. Patients were determined to be ‘cured’ if they had completed treatment and had at least three consecutive negative sputum smears for AFB, with at least 30 days in between; ‘treatment completion’ was attributed to patients having completed treatment but without bacteriologic confirmation; a label of ‘death’ was given to patients having died for any reason during the course of treatment; ‘treatment failure’ was considered in patients with a positive sputum smear for AFB at 5 months or later after initiation of MDR-TB regimen or bacteriological reversion to positive after conversion (relapse). ‘LTFU’ referred to patients lost to follow-up for two or more consecutive months. In the present study, we defined ‘successful treatment’ for patients declared cured, and ‘unsuccessful treatment’ for all other patients, i.e. those who died, failed treatment, relapsed, completed treatment without proof of cure, or were lost to follow-up.

### Data collection and biomarkers of ageing assessments

#### Personal data

Using a face-to-face standardized questionnaire, trained health workers obtained sociodemographic data and information on lifestyle and occupational history (specifically related to past or present mining-related activities). A positive history of mining was considered in participants reporting current or recent (< 2 months) work in mining (digging) or ancillary activities (ore washing, sifting); children present at work with their mother (and possibly helping) were also considered as involved in mining activities with duration referred to that of their mother.

#### Specimen collection

At recruitment, research nurses collected venous blood from a brachial vein into a 4-ml vacutainer tube spray-coated with K_2_EDTA (BD367844) using a butterfly Valve Set G23 (BD387435). A urine sample was also obtained for measurements of trace metals but these data will be presented elsewhere. The samples were first stored at − 18 °C, then shipped to Belgium in cool boxes as checked luggage, and finally stored at − 20 °C prior to analysis (Supplementary Table 1).

### Average relative leukocyte telomere length and mtDNA content

Peripheral whole blood leukocyte DNA was extracted using the QIAamp DNA Blood Mini Kit (Qiagen, Inc., Venlo, the Netherlands). DNA quantity and purity were assessed by a Nanodrop 1000 spectrophotometer (Isogen, Life Science, Belgium). DNA was considered pure when the A260/280 was greater than 1.80 and A260/230 greater than 2.0. DNA integrity was assessed by agarose gel electrophoresis. Both TL and mtDNA content were measured using previously described and validated methods^23,35^. All measurements were performed in triplicate on a 7900HT Fast Real-Time PCR System (Applied Biosystems) in a 384-well format. After each qPCR, a melting curve analysis was performed. For each run, a 6-point serial dilution of pooled DNA was performed to assess PCR efficiency and two inter-run calibrators were used to account for inter-run variability. qPCR curves for each sample were visually inspected; when technical problems were detected or high variability was seen among triplicates, samples were removed for further analysis (n = 1 for TL and n = 2 for mtDNA). The relative average TL and mtDNA content were calculated using qBase software (Biogazelle, Zwijnaarde, Belgium). We expressed TL as the ratio of telomere copy number to single-copy gene number (T/S) relative to the average T/S ratio of the entire sample set (n = 122). Similarly, mtDNA was expressed as the ratio of ND1 copy number to single-copy gene number relative to the average ratio of the entire sample set (n = 121). The coefficients of variation (CV) for triplicates of the telomere runs, ND1 runs, single-copy gene runs, T/S ratios, and mtDNA content were 0.46%, 0.45%, 0.31%, 6.9%, and 6.7%, respectively.

### Statistical analyses

We excluded eight persons from statistical analyses because of insufficient blood for determining TL (five samples) or mtDNA content (six samples), and because of a failed DNA quality check (2 samples). We summarized participant characteristics using mean and standard deviation (SD) or median and interquartile range (IQR) for continuous variables, and frequency for categorical variables. Chi-square and two-sample independent t-test (Mann–Whitney tests) were used as appropriate for group comparison. Due to their skewness, TL and mtDNA content were log-transformed. We compared the outcomes of TB treatment (successful vs unsuccessful treatment) between participants with a history of mining and those never involved in mining. We also evaluated the relations between blood TL or mtDNA content and outcome of TB treatment. We used multivariable linear regression to assess predictors of biomarkers of ageing. Cox regression modelling identified predictors independently associated with treatment success or failure for the entire group and after stratifying by history of mining. All models were first adjusted for demographic variables (sex and age) and then to other known covariates likely to impact the outcomes such as body mass index (BMI), health-related behaviors (e.g., tobacco smoking), length of time spent in mining-related job, history of HIV infection and type of TB (new case vs retreatment). All P values are 2-sided. All statistical analyses were performed using Stata SE 14.0 statistical software (Stata Corporation, College Station, TX, USA). Graphs were made using GraphPad Prism 8.1.2 Software (GraphPad Software, San Diego, CA, USA).

### Ethics approval and consent to participate

The protocol was approved by the ethics committee of the Catholic University of Bukavu in the frame of the “Environmental Exposure and Risk of Respiratory Illnesses in Kivu (EERRIK Project)” (UCB/CIE/NC/01/2018). Letters of support were obtained from the South Kivu Provincial Health Department regulatory authorities (South Kivu Health Department and Provincial Programme for Tuberculosis). Informed consent to publish identifying information/image was obtained. All the participants provided their written informed consent before participating in the study and all research was performed in accordance with relevant guidelines/regulations.

## Supplementary Information

Supplementary Table 1.
