# Supplementary material for: Telomere length and outcome of treatment for pulmonary tuberculosis in a gold mining community
Source: Sci Rep. 2021 Feb 17;11:4031. doi: 10.1038/s41598-021-83281-2 (PMC7889934; doi:10.1038/s41598-021-83281-2)
Supplement: Supplementary file 1 — Supplementary Table 1. [file 41598_2021_83281_MOESM1_ESM.pdf]

Telomere Length and Outcome of Treatment for Pulmonary Tuberculosis in a Gold Mining Community

Patrick D.M.C Katoto[1,2,3], Tony Kayembe-Kitenge[1,4], Krystal J. Godri Pollitt[5], Dries S. Martens[6], Manosij Gosh[1], Jean B. Nachega[3,7,8], Benoit Nemery[1], Tim S. Nawrot[1, 6]

Online Data Supplement

Table E1. Multivariable Linear Regression Analysis of Predictors for Leukocyte Telomere Length (Including Treatment Outcomes)

Variables	Adjusted Model											
	All Participants				Mining-Related Job							
					No				Yes			
	Coef·	[95% Conf Interval]		p-value	Coef·	[95% Conf Interval]		p-value	Coef·	[95% Conf Interval]		p-value
**Mining-related-activities**												
No	Ref.											
Yes	-0·102	-0·199	-0·006	0·038	—	—	—	—	—	—	—	—
**Age (+1 year)**					0·086	-1·12	1·29	0·852	-0·160	-0·351	0·032	0·10
**Sex**												
Female					Ref.							
Male					0·254	-0·903	1·41	0·576	0·119	-0·017	0·254	0·08
**BMI (+1kg/ m^2)**					1·06	-2·79	4·98	0·486	0·319	-0·189	0·827	0·21
**Tobacco-Smoking**												
No					Ref.							
Yes					-0·463	-2·36	1·43	0·535	-0·157	-0·364	0·050	0·13
**New Case**												
New case					Ref.							
Retreatment					0·352	-1·31	2·02	0·589	-0·131	-0·293	0·031	0·11
**HIV(+)**												
**No**					Ref.							
**Yes**					0·099	-0·739	0·94	0·759	-0·186	-0·347	-0·025	0·025
**Cured**												
**Yes**					Ref.							
**No**					0·122	-0·992	1·24	0·777	0·153	-0·002	0·308	0·052
**Mining**					—	—	—	—	-0·044	-0·129	0·041	0·30

BMI: body mass index· Ref·: reference· All models are adjusted for sociodemographic and clinical variables·
